# Response to school‐based interventions for overweight and obesity: A systematic scoping review

**DOI:** 10.1111/cob.12557

**Published:** 2022-09-21

**Authors:** Katherine R. Arlinghaus, Aliye B. Cepni, Rachel R. Helbing, Lenora P. Goodman, Tracey A. Ledoux, Craig A. Johnston

**Affiliations:** ^1^ Division of Epidemiology and Community Health, School of Public Health University of Minnesota Minneapolis Minnesota USA; ^2^ Department of Health and Human Performance University of Houston Houston Texas USA; ^3^ University Libraries University of Houston Houston Texas USA

**Keywords:** children, community‐based, nutrition, response heterogeneity

## Abstract

Heterogeneity of response to paediatric obesity interventions is one of the greatest challenges to obesity care. While evaluating school‐based interventions by mean changes compared to control is important, it does not provide an understanding of the individual variability in response to intervention. The objective of this study was to comprehensively review school‐based interventions that reported study results in terms of response and identify definitions of response used. A scoping review was conducted using a systematic search of five scientific databases from 2009 to 2021. Inclusion criteria included randomized controlled trial design, school‐based setting, weight‐based outcomes (e.g., BMI, BMI z‐score), weight‐based outcomes analysed among youth with overweight/obesity, a study conducted in a developed country and publication in English. A total of 26 reports representing 25 unique studies were included. Overall, 19% (5/26) of articles reported response. Response was defined in three ways: maintenance/decrease in BMI z‐score, decrease in BMI z‐score ≥0.10, and decrease in BMI z‐score ≥0.20. Few school‐based interventions identified an a priori intervention goal or identified the proportion of participants who responded to the intervention. Without such evaluation participants who do not benefit are likely to be overlooked.

## 1 INTRODUCTION

The scope and severity of childhood obesity are well documented, warranting the prevalence of obesity to be referred to as an ‘epidemic’ and a ‘public health crisis’.^1^ Evaluation of paediatric obesity intervention traditionally relies on comparing mean changes between intervention and control groups.^2^ This programmatic evaluation has played an important role in improving paediatric obesity intervention. For example, although schools were identified as an important focal point for intervention,^3^ initially many school‐based interventions did not improve child weight‐related outcomes.^4^, ^5^ Through comparing mean changes between intervention and control conditions, intervention programs were revised and the effectiveness of school‐based interventions has improved.^6^

Despite the benefit of comparing mean changes between groups, real world conditions of community‐based and public health interventions do not have control groups to which intervention outcomes can be compared. Identifying an *a priori* goal for intervention response is important to determine intervention utility and if an intervention is having a meaningful impact. This approach is important at all stages of intervention research and is consistent with the ORBIT model which emphasizes the need for preliminary evidence that intervention outcomes are likely to have clinically meaningful outcomes before doing larger scale efficacy trials.^7^ Similarly, understanding the proportion of participants who reach clinically meaningful outcomes can help identify which interventions should be disseminated as well as to track outcomes of an intervention once disseminated. For example, changes in the proportion of participants who reach clinically meaningful outcomes overtime from an intervention could signal issues with implementation fidelity.

Currently, there is no agreed upon definition or criteria for clinically meaningful response to paediatric obesity interventions.^8^ An increasing number of weight loss interventions among adults report the proportion of individuals who achieve the 5%–10% threshold of weight loss associated with cardiometabolic improvements.^2^ This type of evaluation parallels the efficacy standards needed for the FDA to approve a weight loss drug. Specifically, at least 35% of individuals taking the drug must reach ≥5% weight loss for the drug to be considered effective.^9^ While not used as an a priori intervention goal or evaluation metric, a wide range of paediatric response definitions have been developed out of necessity as part of secondary analyses investigating characteristics predictive of response in clinic‐based paediatric intervention. Definitions used include the maintenance or decrease in standardized body mass index (zBMI)^10^, ^11^, ^12^ or BMI represented as a percentage of the 95th BMI Percentile,^13^ ≥5% reduction in zBMI,^14^, ^15^, ^16^ ≥10% reduction in zBMI,^16^ ≥0.20 reduction in zBMI,^17^ or a 5% reduction in weight.^18^ Response time frames ranged from post intervention^16^, ^18^ to 2 years follow‐up,^13^, ^16^, ^17^ with most studies analysing response at 1 year.^10^, ^11^, ^13^, ^14^, ^15^

The purpose of this scoping review was to (1) identify the proportion of school‐based interventions that report study results in terms of response and to (2) examine definitions used for response to school‐based intervention. Understanding how response to school‐based intervention is evaluated and reported is particularly important as there is likely to be greater variation in response to school‐based intervention than to clinical interventions among only treatment‐seeking participants. Similarly, understanding how many students reach meaningful improvements from school‐based intervention is important to determine if the intervention should be continued and/or disseminated further. With school‐based interventions as an example, the findings from this review will shed light on the current state of the literature with regards to how meaningful change is being defined and reported in community‐based paediatric obesity interventions.

## 2 METHODS

### 2.1 Study design

This scoping review intended to analyze the state of current literature on school‐based obesity interventions, particularly how response is defined and reported, which falls under Kirksey and O′Malley's first scoping review purpose, “to examine the extent, range and nature of research activity.”^19^ The review is being reported according to the Preferred Reporting Items for Systematic Review and Meta‐Analyses Extension for Scoping Reviews (PRISMA‐ScR).^20^

### 2.2 Search strategy

Systematic searches, developed by a health sciences librarian in collaboration with subject experts, were conducted in PubMed, Scopus, PsycInfo (EBSCO), Education Source (EBSCO), and ERIC (EBSCO) on 12 August 2021. A comprehensive list of keywords and subject terms covering the concepts of school‐based programs, body mass index, and children and adolescents were combined using the appropriate Boolean operators. Additionally, filters for study type (randomized controlled trials), publication date (2010—date of search), language (English), and geographic region (exclusion of Africa, Antarctic and Arctic regions, and Asia) were applied. Complete search strategies are included in supplement information S1.

### 2.3 Inclusion/exclusion criteria

Inclusion criteria were a school‐based setting, an outcome measuring weight or BMI, school‐aged paediatric participants, randomized controlled trial, and publication in English in 2010 or later. With this being the initial review to examine how response to intervention was being reported, it was important to include only randomized controlled trials to keep the data synthesis as clean as possible. Included articles were limited to those published in 2010 or later because the focus on precision medicine and heterogeneity in response to obesity intervention is relatively new among paediatric populations.^2^ Accordingly the most relevant articles are likely to be captured in the past decade of research. Exclusion criteria were any settings outside of school such as churches or community centres, pre‐school settings, primary prevention studies or studies that did not measure weight or BMI, non‐randomized controlled trial study designs, publication in languages other than English, and locations in Africa, Antarctic or Arctic regions, or Asia. These geographic region exclusions were applied to primarily limit results to westernized nations. Articles were also excluded if they assessed weight‐based outcomes on samples with mixed weight statuses (i.e., only articles with an analysis for ‘secondary prevention’ populations were included).

### 2.4 Article screening

**FIGURE 1:**
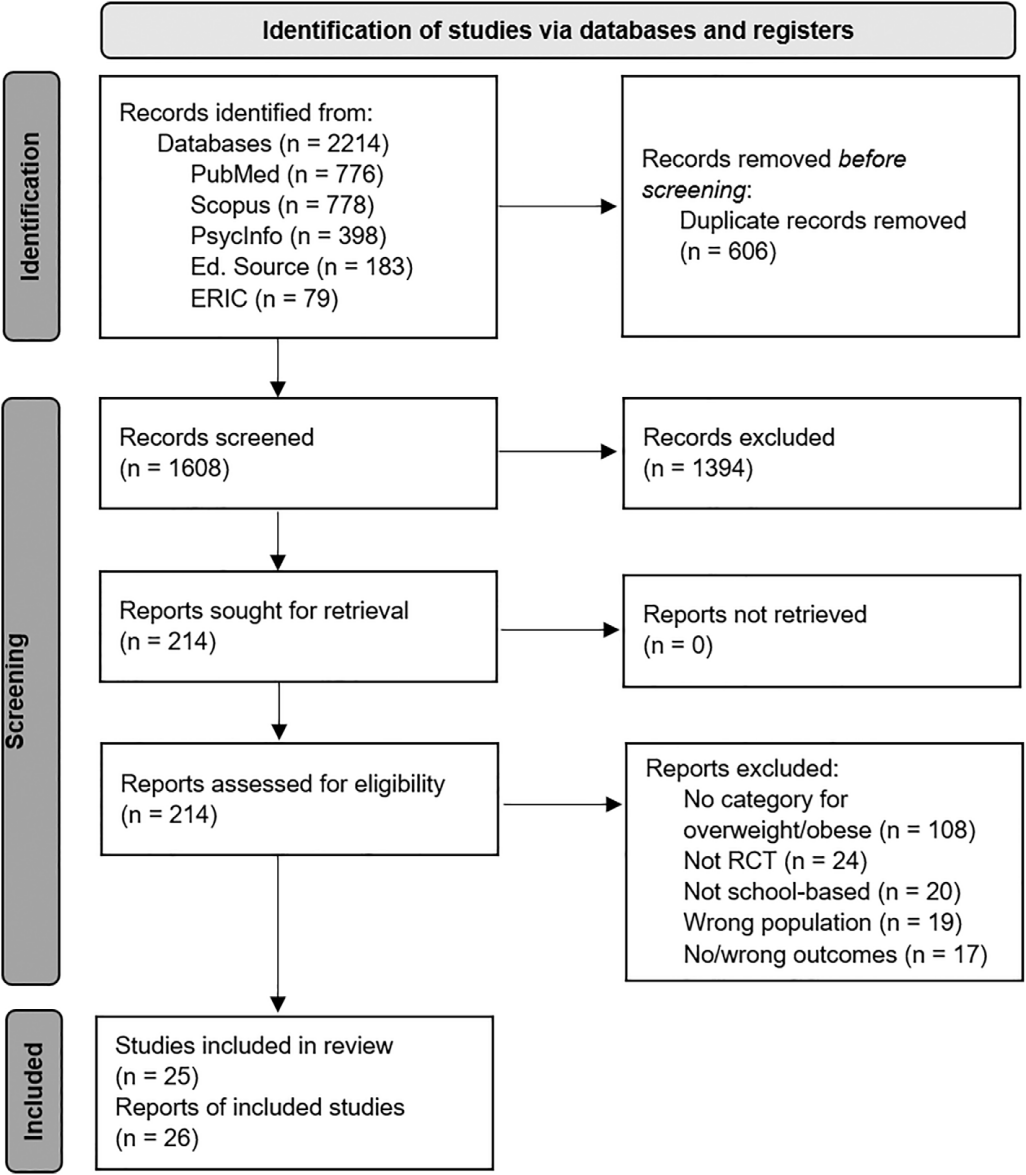
Flow diagram.

The database searches yielded a combined 2214 records. A first round of deduplication was conducted in EndNote, and a second round was done in Rayyan, a free web‐based systematic review screening tool developed by the Qatar Computing Research Institute.^21^ Rayyan was then used to screen the remaining 1608 unique records for inclusion and exclusion. For the first level of screening, two researchers reviewed the titles and abstracts of the citations to eliminate articles that did not meet the minimum inclusion criteria. When opinions between these two researchers differed, a third researcher reviewed the article. Reviewers met to resolve any conflicts and ensure consistency. Disagreements were resolved by a majority vote. This same process was followed for the full‐text review of articles, and articles not meeting eligibility criteria were excluded. Reasons for exclusion were documented within Rayyan. See Figure 1 for the PRISMA flow diagram.

### 2.5 Data extraction

The characteristics of each article were extracted by two reviewers. Reviewers extracted the following information regarding the reporting of response: was any definition of response reported (yes or no), definition of response if it was reported, was the difference in the proportion of participants who met the response definition tested between intervention conditions (yes or no), and the response outcome reported. Specifically, the term ‘response’ refers to a threshold of meaningful change in weight outcomes as defined and reported (or not) in each study. After researchers independently extracted data, it was compiled into a single spreadsheet. Data synthesis was performed through researcher discussion examining the similarities and differences of each study. As part of this discussion, tables were created to map out the characteristics of the study population (Table 1), intervention description and outcomes (Table 2), and response reporting (Table 3). These tables were used to identify patterns in how meaningful change is being defined and reported in school‐based obesity interventions.

**TABLE 1:** Description of participant characteristics of included studies

Study (first author's last name and year published)	Study location	Participant characteristics					
		Age (years) at baseline, *mean (SD)*	Gender	Race/Ethnicity	Income	Baseline BMI‐based measure, *mean (SD)*	Sample size, *Total: Intervention/Control*
Adab 2018^26^	UK (West Midlands)	6.29 (0.31)	48.9% Female	45.3% White British 30.5% South Asian 7.9% Black African Caribbean	54.9% IMD quintile of 1 (most deprived)	zBMI Int–1.99 (0.81) Cont–1.94 (0.73)	54 schools: 26/28 252: 124/128
Arlinghaus 2017^35^	USA (Houston, TX)	Int–12.91 (0.48) Cont–12.94 (0.63)	% Female: Int–51% Cont–52%	100% Hispanic	NR	zBMI: Int–1.78 (0.41) Cont–1.76 (0.46) BMI percentile Int–95.04 (3.82) Cont–94.57 (4.27) BMI: Int–27.4 (4.03) Cont–27.51 (4.53)	189: 94/95
Arlinghaus 2019^34^	USA (Houston, TX)	12.02 (0.57)	50.7% Female	100% Mexican‐American	NR	zBMI: 1.80 (0.46) BMI percentile: 94.93 (4.18) BMI: 27.03 (4.95)	243: 1 day/week = 59, 3 days/week = 58, 5 days/week = 63, control/0 days/week = 63
Arlinghaus 2021^38^	USA (Houston, TX)	12.1 (0.63)	53% Female	100% Hispanic‐American	NR	zBMI: 0.89 (1.09) BMI percentile: 72.21 (28.03) BMI: 22.64 (5.75)	491: 251/240
Bogart 2016^37^	USA (Los Angeles, CA)	7th grade 12.2 (0.68)^a^	50.9% Female^a^	75.4% Latino^a^ 60.7% US‐born	88.9% free/reduced participation in NSLP (FRL)^a^	BMI percentile: *Overweight* Int–90.60 (2.94) Cont–91.22 (2.74) *Obesity* Int–97.92 (1.24) Cont–97.67 (1.30)	*Overweight*: 275:NR *Obesity*: 415:244/171
Daly 2016^41^	USA (AZ)	Int–15.4 (1.4) Cont–15.6 (0.9)	100% Female	100% Latinas	NR	BMI Int–37.7 (7.6) Cont–34.3(6.2)	37:14/23
Davis 2021^31^	USA (Austin, TX)	9.23 (0.02)	52.65% Female	63.67% Hispanic	68.25% FRL eligible	zBMI: Int–0.77(0.09) Cont–0.81(0.08) BMI percentile: Int–69.99(2.18) Cont–71.05(1.99) BMI: Int–20.03(0.35) Cont–20.08(0.31)	16 schools: 8/8 3135: 1412/1723
Foster 2010^36^	7 field sites across USA	6th grade Int–11.2 (0.5) Cont–11.3 (0.6)	% Female: Int–49.5% Cont–49.6%	Hispanic: Int–58.4% Cont–56.7% Black: Int–18.4% Cont–16.2% White: Int–15.5% Cont–18.6%	NR	zBMI: 1.80 (0.44)	42 schools: 21/21 2292:1160/1132
Johnston 2010^32^	USA (Houston, TX)	6th & 7th grade 12.3 (0.7)	% Female: Int–47.5% Cont–40%	100% Mexican‐American	NR	zBMI: Int–1.5 (0.6) Cont–1.7 (0.6) BMI: Int–25.2 (4.4) Cont–26.7 (5.5)	60: 40/20
Johnston 2013a^27^	USA (Houston, TX)	2nd grade Int–7.8 (0.4) Cont–7.7 (0.4)	% Female: Int–38.2% Cont–45.9%	Asian Int–24.2%, Cont–16.3% Black Int–26.9%, Cont–26.7% Hispanic Int–27.4% Cont–29.6% White Int–21.5%, Cont–27.4%	NR	zBMI: Int–1.8 (0.5) Cont–1.7 (0.4) BMI: Int–21.6 (3.9) Cont–21 (2.6) BMI percentile: Int–94.5 (4.5) Cont–94.7 (3.9)	321: 186/135 7 schools: 4/3
Johnston 2013b^33^	USA (Houston, TX)	6th & 7th grade 2.54 (0.55)	54.9% Female	100% Mexican‐American	NR	zBMI: Int–1.9 (0.5) Cont–1.6 (0.4) BMI: Int–27.7 (5) Cont–25.6 (3.4)	71: 46/25
Jones 2015^24^	Australia (Wollongong)	*Girls*: 9.6 (0.8) *Boys*: 10.2 (0.8)	45.9% Female	*Girls* 76% Australian 6% Asian *Boys* 85% Australian 10% Asian	Low income; 56.8% < AUD $60 000	zBMI: *Girls* Int–2.1 (0.8) Cont–2.4 (0.8) *Boys* Int–1.9 (0.9) Cont–2.5 (1.0) BMI: *Girls* Int–22.9(3.5) Cont–23.2 (3.8) *Boys* Int–22.9 (3.2) Cont–26.03 (4.5)	37: 19/18 *Girls* 17:9/8 *Boys* 20: 10/10
Kong 2013^42^	USA (NM)	Int–15 (1.0) Cont–14.6 (0.7)	58.8% Female	68.6% Hispanic 9.8% Asian 5.9% Native American	NR (most SBHCs serve predominately low income students)	BMI percentile: Int–94.5 (4.1) Cont–94.4 (4.6)	2 schools: 1/1 60: 31/29
Kubik 2021^30^	USA (MN)	9.3 (0.9)	49% Female	23% Hispanic 21% Black 37% White 19% Other	59% Economic assistance	zBMI: 1.6 (0.7) BMI: 23.0 (5.0)	132 (66/66)
Love‐Osborne 2014^43^	USA (CO)	Int–15.7 (1.5) Cont–16.0 (1.5)	% Female Int–58% Cont–46%	% Hispanic: Int–88% Cont–89%	NR	BMI: Int–31.9 (6.2) Cont–31.6 (6.5) zBMI: Int–1.92 (0.46) Cont–1.89(0.52)	2 schools: 1/1 165:82/83
Mabli 2020^39^	USA (NY)	13.6 (1.9)	53.4% Female	NR	NR	zBMI: 1.61 (0.40)	436: 233/203
Madsen 2021^47^	USA (CA)	3rd through 7th grades^a^	48.9% Female^a^	58.9% Hispanic 7.5% African American 15.5% White 15.1% Asian^a^	68.4% FRL^a^	zBMI: 0.61 (1.15)^a^	79 schools: 27 (Intense int) /27 (int)/25 (cont) 28 641:10041/10441/8159^a^
Smith 2014^22^ Lubans^23^, ^a^	Australia (New South Wales, Wollongong)	12.7 (0.5)^a^	100% Male	77.2% Australian 14.8% European 1.9% African 1.9% Asian^a^	Schools all located in Socioeconomic Indexes For Areas (SIFA) ≤5 (lowest 50%)^a^	BMI: 20.5 (4.1)^a^	14 schools 361: 181/180^a^
Pbert 2013^44^	USA (MA)	Int–15.9 (1.03) Cont–15.7 (1.01)	% Female Int–64.3% Cont–75.0%	White: Int–73.8% Cont–80.0% Black: Int–14.3% Cont–5.0% Hispanic: Int–14.3% Cont–15.0%	FRL: Int–47.6% Cont–17.5%	BMI: Int–32.8 (5.91) Cont–31.2 (5.31) zBMI: Int–1.95 (0.44) Cont–1.81 (0.41)	6 schools: 3/3 82: 42/40
Pbert 2016^45^	USA (MA)	Int–16.5 (1.23) Cont–16.3 (1.20)	% Female: Int–63% Cont–61.4%	White Int–63% Cont–63.2% Black Int–24.1% Cont–15.8% Hispanic Int–24.1% Cont–38.6% Mixed Int–5.6% Cont–21.1%	FRL: Int–51.9% Cont–63.2%	BMI: Int–30.7 (5.35) Cont–31.6 (5.20) zBMI: Int–1.7 (0.52) Cont–1.9 (0.48)	8 schools: 4/4 126: 58/68
Robbins 2020^40^	USA (Midwest)	12.07	100% Female	61.3% Black	78.6% low SES	zBMI: Int–1.30 (0.74) Cont–1.42 (0.73)	24 schools: 12/12 1194: 593/601
Santos 2014^25^	Canada (Manitoba)	Int–9.3 (95 CI: 9.1–9.5) Cont–8.8 (95 CI: 8.6–9.0)^a^	48% Female^a^	28% First nations (indigenous)^a^	NR	zBMI: Int–0.64 (95 CI: 0.52–0.76) Cont–0.55 (95 CI: 0.42–0.68)^a^	20 schools: 10/10 Younger students 108: 55/53 Older students 131: 74/57
Staiano 2013^46^	USA (WA)	15–19	55.6% Female	100% African‐American	NR	BMI percentile: 94.7 (6.0)	54: 19 competitive exergame, 19 cooperative exergame, 16 control
Williamson 2012^29^	USA (LA)	10.5 (1.2)^a^	58.5% Female^a^	68.4% African‐American 31.6% White^a^	77.0% FRL^a^	zBMI: 0.78 (1.16)^a^ BMI Percentile: 69.7 (29.5)	17 schools: primary prevention 5/primary + secondary prevention 6/ control 6 2060: 713/760/587^a^
Wright 2012^28^	USA (Los Angeles, CA)	Int–9.0 (1.6) Cont–8.3 (1.1)	% Female Int–58% Cont–62%	Mexican‐American Int–96% Cont–95%	$0‐15 K: Int–56% Cont–61% $15‐25 K: Int–44% Cont–39%	BMI: Int–21.89 (6.26) Cont–21.25 (6.68) zBMI: Int–2.30 (0.41) Cont–2.28 (0.50)	5 schools: 2/3 305: 165/140

Abbreviations: Cont, control group; FRL, eligible for free/reduced school lunch in national school lunch program; Int, intervention group; IMD, indices of multiple deprivation, assessed using postal code; zBMI, standardized body mass index.

^a^ Includes participants with ‘primary prevention’ goals (healthy weight status) because descriptors of only the participants with a ‘secondary prevention’ goal were not provided. Secondary prevention populations were generally defined as a BMI percentile ≥85, but some studies also included those at risk of overweight defined as a BMI percentile ≥75^24^, ^30^, ^46^ or zBMI >1.0.^40^ Lack of superscript ‘a’ indicates descriptor of only those in the “secondary prevention population”.

**TABLE 2:** Study characteristics and primary outcomes of included studies

Study (first author's last name and year published)	Study design (randomization level)	Theoretical framework	Intervention duration	Description of study conditions		Primary weight‐based outcome
				Intervention	Control	
Adab 2018^26^	RCT (school‐level)	Developed from Medical Research Council framework	1 year (outcomes reported at 3 months post intervention, 18 and 27 months)	Multicomponent including signposting, PA, cooking workshops, healthy lifestyle challenges in community program run by a English Premier League football club.	Control schools provided resources unrelated to health or healthy lifestyle behaviours.	Changes in zBMI were not significantly different between int and control.
Arlinghaus 2017^35^	RCT (individual‐level)	NR	6 months (outcomes reported at 6 months and 1 year)	High school students trained as peer‐health mentors to middle school students during PE class. High school students completed all activities with middle school students. Weekly PE classes included 1 day nutrition & 4 days PA.	Same PE class as intervention (1 day of nutrition, 4 days PA) but without peer mentors.	Mean zBMI reduced significantly more in intervention than control (at 6 and 12 months).
Arlinghaus 2019^34^	RCT (individual‐level)	SCT	24 weeks, outcomes reported at 1 year	Three intervention arms based on the frequency of intervention provided (5 days, 3 days, or 1 day per week). Intervention provided in PE class, 80% of time was spent on PA, and 20% on nutrition.	0 days/week (no PE class)	zBMI reduced significantly more in 5 days/week and 3 days/week compared to control. No difference between 5 and 3 day/week groups.
Arlinghaus 2021^38^	RCT (individual‐level)	SCT	6 months	Intervention provided in PE class (5 days/week). Classes designed to provide facilitate MVPA. Lessons were circuit‐based and included aerobic and strength training exercises. No nutrition.	5 days/week, PE class as usual (sports‐based units)	Among those with obesity, intervention had significantly greater decreases in zBMI than control. No difference between conditions among overall sample.
Bogart 2016^37^	RCT (school‐level)	NR	5‐week intervention, 2 year outcomes reported	SNaX combined school‐wide environmental changes (healthier food, water availability, posters promoting desired health behaviours) with peer‐led education about healthy eating and PA. Participants received weekly activities to do with parents.	NR	Among those with obesity, intervention had significantly greater decreases in BMI Percentile than control. No difference between conditions among those with overweight.
Daly 2016^41^	Feasibility RCT (individual‐level)	Information‐Motivation‐Behavioural Skills Theory	6 weeks intervention, 10‐week outcomes reported	Weekly 90 min mindful eating sessions included mindfulness meditation, instruction, discussion, and eating skills practice. Satiety awareness was practiced with group activity rating hunger before and after traditional Mexican meal.	One‐time receipt of written nutrition and PA information	Intervention reduced BMI significantly more than control at 6 weeks. Intervention continued to decrease BMI at 10 weeks.
Davis 2021^31^	RCT (school‐level)	Social Ecological‐Transactional Model	9 months	Lessons designed to improve diet‐related psychosocial constructs, including increasing nutrition, gardening, and cooking knowledge, self‐efficacy and attitudes, and willingness to try and preference for FV, and reducing food insecurity.	Control schools received delayed intervention a year after post‐testing.	The intervention did not have an effect on any BMI parameters.
Foster 2010^36^	RCT (school‐level)	NR	3 years	Integrated nutrition (quantity/quality of school food), PA (increase MVPA during PE class), behavioural skills (classroom program to increase knowledge, self‐monitoring, self‐awareness, goal setting), and communications/social marketing.	NR	Students in intervention schools had 21% lower odds of having obesity at the end of 8th grade than in the control schools.
Johnston 2010^32^	RCT (individual‐level)	NR	24 weeks (3 and 6 month outcomes previously published, reports 1 and 2 year outcomes)	5 days/week, instructor‐led healthy eating and physical activity intervention during last school period; (specific intervention content not provided, referenced primary outcomes manuscript),	Study hall and provided parent‐guided manual for child weight loss	Mean reduction in zBMI among intervention > than control at 1 and 2 years. Significantly more participants maintained/decreased zBMI at 1 year in the intervention than control group.
Johnston 2013a^27^	RCT (school‐level)	NR	NR (outcomes reported at 1 and 2 years)	Materials provided to control schools + health professional provided technical assistance and motivational interviewing to staff to assist with sustained implementation	Curriculum materials with integrated health information, teaching aids, and educational materials provided to teachers	Mean reduction in zBMI among intervention > than control at 2 years (no significant difference between groups at 1 year),
Johnston 2013b^33^	RCT (individual‐level)	NR	24 weeks (3 and 6 month outcomes previously published, reports 1 and 2 year outcomes)	Culturally tailored nutrition (1 day/week) and PA (4 days/week). Trained undergraduate students assisted with lessons. Monthly parent meetings.	Provided parent‐guided manual on child weight management.	Mean reduction zBMI among intervention > control at 1 and 2 years. Significantly more adolescents in intervention reduced/maintained zBMI at 2 years than control.
Jones 2015^24^	Pilot RCT (individual‐level)	SCT	7 months, 7 and 12 month outcomes reported	Biweekly gender‐tailored afterschool PA programs included 30 min homework and 90 min structured PA designed to optimize MPVA time. Weekly at‐home challenges paired with a reward system.	Weekly afterschool sessions: 30 min homework, 45 min classroom healthy lifestyle education and 45 min PA focused on light PA.	At 7 months intervention girls had greater improvements zBMI and body fat % than comparison. Intervention boys had greater improvements in waist circumference z‐score. Improvements were not maintained at 12 months for boys or girls.
Kong 2013^42^	Feasibility RCT, (school‐level)	Transtheoretical Model	1 academic year (~9 months)	Clinical encounters with SBHC nurse every 2–3 weeks (8 visits total). Nurse used motivational interviewing and obesity risk reduction strategies from a toolkit developed by community advisory group. Newsletter sent home with participants and caregivers were given telephone updates after each session.	Standard SBHC care with family medicine physician including 1 clinic visit at the beginning of the trial, the AAP ‘Balance for a Healthy Life’ booklet, and medical results summary with AAP recommendations.	Intervention participants had greater improvements in BMI Percentile and Waist circumference than standard care group.
Kubik 2021^30^	RCT (individual‐level)	Social–Ecological Framework & Healthy Learner Model	9 months (12 and 24 month outcomes reported)	School nurses delivered 90‐min group sessions including food preparation by the child, hands‐on activities, games, and goal setting linked to the behavioural message and 30 min of PA. 90‐min parent group sessions included dinner ideas, activities with behavioural messages consistent with kid group themes, and 30 min of PA. 60‐min home visit focused on tailored family goals for behaviour change.	Monthly newsletter with family‐oriented healthy lifestyle information (bike and car safety and family first aid, recipes, community events)	No significant difference in zBMI and BMI between conditions at 12 and 24 months.
Love‐Osborne 2014^43^	Feasibility RCT (school‐level)	NR	Academic school year	SBHC with health educator that reviews goals using MI and incentivized participants to self‐monitor. Educator encouraged participants to choose 1 nutrition goal and 1 PA goal, provided resources, and electronic support through text messages.	SBHC care as usual.	Significantly more participants in SBHC care as usual had a significant decrease in zBMI than intervention with health educator (40.3% compared to 18.2%).
Mabli 2020^39^	RCT (individual‐level)	NR	12 weeks	Get Fit was an intensive program in which students identified and worked on specific goals for im‐ proving their eating or physical activity habits and improving their health. Families of students participated in cooking demonstrations and education workshops.	Prevention only group included healthy snacks and opportunities to engage in MVPA/day after‐school programming, and parent nutrition workshops.	Intervention significantly decreased zBMI compared to control among girls, but not boys.
Madsen 2021^47^	3‐arm RCT (school‐level)	NR	Yearly screening	Group 1: BMI screened by school staff and reported to parents. Group 2: BMI screened by school staff but not reported to parents.	No BMI screening or reporting.	No difference in zBMI change between group 1 and 2 students at 1 year and at 2 years.
Smith 2014^22^ Lubans 2016^23^	RCT (school‐level)	Self‐determination theory & SCT	20 weeks, 8 month^22^ and 18 month^23^ outcomes reported	Multicomponent intervention including teacher professional development, parent newsletters, interactive PA seminars, enhanced school sport sessions, PA mentoring, and self‐monitoring.	Regularly scheduled school sports and PE lessons	No significant intervention effects for BMI or zBMI at 8 months or 18 months; significant within‐group reduction in zBMI observed among intervention group at 18 months.
Pbert 2013^44^	Feasibility RCT (school‐level)	SCT	2 months (2 and 6 month outcomes reported)	6 individual nurse‐led counselling sessions: encouraged PA and diet changes, cognitive behavioural techniques to develop self‐management behaviours.	6 individual nurse visits: weight assessment, weighed, review behaviour changes, and informational weight management pamphlets.	No statistically significant differences between conditions in zBMI or BMI at 2 or 6 months.
Pbert 2016^45^	RCT (school‐level)	SCT	8 months	6‐weekly 30‐min individual sessions followed by 6 monthly sessions and weekly weigh‐ins. Sessions included weigh in, diet and PA log review, assessment of progress and troubleshooting, weekly health topic, goal setting. Participants given pedometer. Exercise program 3 sessions/week for 8 months (group games and fitness activities).	12 individual visits with school nurse including weight assessment, review behaviour changes, and read weight management pamphlets	No significant differences in BMI over the course of the intervention between conditions.
Robbins 2020^40^	RCT (school‐level)	NR	17‐weeks	‘Afterschool PA program’, intervention details not reported.	Usual activities	No differences in zBMI overtime between conditions, intervention increased %body fat less than control.
Santos 2014^25^	RCT (school‐level)	NR	~10 months (1 school year)	Older classes (grades 4–6) paired with younger classes (grades K‐3). Each week older students received 45 min lesson from classroom teacher focused on PA, nutrition, and body image. That same week older students taught a 30‐min lesson to younger students. Two lessons per week included structured aerobic fitness.	Wait list control	Significant intervention effect for waist circumference and zBMI among younger children (grades K‐3). Significant intervention effect for waist circumference among older children (grades 4–6).
Staiano 2013^46^	RCT (individual‐level)	SCT	20 weeks	Encouraged to play Nintendo Wii Active game for 30–60 min/day (at lunch or afterschool). Randomized to cooperative (work with peer to earn points) or competitive play (compete against peer).	Usual daily activities	Cooperative exergame improved weight significantly more than control. No significant difference between competitive group and other conditions.
Williamson 2012^29^	RCT (school‐level)	NR	18 and 28 month outcomes	Prevention Program (PP) modified environmental cues regarding healthy eating and PA, modified food service, modified PE based on SPARK program. PP+ SP included all of the above plus secondary prevention (SP) efforts including classroom instruction with internet‐based approach including email communication to children and parents.	School as usual, did not receive any of the prevention components.	No significant differences between study arms over time in zBMI or body fat percentage among overweight subsample in either girls or boys.
Wright 2012^28^	Pilot RCT (school‐level)	NR	6 weeks, outcomes reported at 12 months	Weekly 90‐min afterschool sessions with PA, nutrition education, and parental support group. School/community level changes included health services from community clinics, School Health Advisory Council, health professional development for staff, and bimonthly health education newsletter mailed home.	Standard school PA program	Intervention group improved BMI and zBMI significantly more than control group at 12 months.

Abbreviations: AAP, American Academy of Paediatrics; MVPA, moderate‐vigorous physical activity; NR, not reported; PA, physical activity; PE, physical education; RCT, randomized control trial; SCT, Social Cognitive Theory; zBMI, standardized body mass index.

**TABLE 3:** Reporting of response across studies

Study (first author's last name and year published)	Response reported	Definition of response	Response statistically tested	Response outcome
Adab 2018^26^	No	n/a	n/a	n/a
Arlinghaus 2017^35^	Yes	Maintained or decreased zBMI (examined at 6 and 12 months)	no	80% of intervention group compared to 64% of control group met response criteria at 6 months 68% of intervention group compared to 55% of control group met response criteria at 12 months
Arlinghaus 2019^34^	Yes	at least 0.20 decrease in zBMI at 12 months	no	Presented in figure only, exact proportions not reported
Arlinghaus 2021^38^	No	n/a	n/a	n/a
Bogart 2016^37^	No	n/a	n/a	n/a
Daly 2016^41^	No	n/a	n/a	n/a
Davis 2021^31^	No^a^	n/a	n/a	n/a
Foster 2010^36^	No^a^	n/a	n/a	n/a
Johnston 2010^32^	Yes	maintained or decreased zBMI (examined at 1 and 2 years)	yes, chi square	79.5% of intervention group compared to 35.5% of control group met response criteria at 1 year (difference statistically significant) 62.1% of intervention group compared to 35.3% of control group met response criteria at 2 years (difference between groups not reported)
Johnston 2013a^27^	No	n/a	n/a	n/a
Johnston 2013b^33^	Yes	maintained or decreased zBMI (examined at 1 and 2 years)	yes, chi square	68.2% of intervention group compared to 42.9% of control group met response criteria at 1 year (difference not statistically significant) 81.6% of intervention group compared to 35% of control group met response criteria at 2 years (statistically significant difference)
Jones 2015^24^	No	n/a	n/a	n/a
Kong 2013^42^	No	n/a	n/a	n/a
Kubik 2021^30^	No	n/a	n/a	n/a
Love‐Osborne 2014^43^	Yes	0.1 decrease in zBMI at ~10 months	yes, chi square	18.2% of intervention group compared to 40.3% of control group met response criteria at end of academic year (difference statistically significant, in favour of control)
Lubans 2016^23^	No^a^	n/a	n/a	n/a
Mabli 2020^39^	No^a^	n/a	n/a	n/a
Madsen 2021^47^	No	n/a	n/a	n/a
Pbert 2013^44^	No	n/a	n/a	n/a
Pbert 2016^45^	No	n/a	n/a	n/a
Robbins 2020^40^	No	n/a	n/a	n/a
Santos 2014^25^	No	n/a	n/a	n/a
Smith 2014^22^	No^a^	n/a	n/a	n/a
Staiano 2013^46^	No	n/a	n/a	n/a
Williamson 2012^29^	No	n/a	n/a	n/a
Wright 2012^28^	No	n/a	n/a	n/a

Abbreviations: zBMI, standardized body mass index.

^a^ Reports movement between weight classification (e.g., proportion of participants who moved from overweight to healthy weight classification).

## 3 RESULTS

A total of 26 records, representing 25 unique studies, met the inclusion/exclusion criteria to be included in this scoping review. Table 1 provides a description of participant characteristics across studies. Most studies (21/25) were conducted in the United States. Two studies occurred in Australia,^22^, ^23^, ^24^ one in Canada,^25^ and one in the United Kingdom.^26^ The age of study participants varied with 8/25 studies having a mean participant age of 10 years or younger (~ elementary school),^24^, ^25^, ^26^, ^27^, ^28^, ^29^, ^30^, ^31^ 10/25 with mean participant age 11–14 years (~middle school),^22^, ^23^, ^32^, ^33^, ^34^, ^35^, ^36^, ^37^, ^38^, ^39^, ^40^ 6/25 with a mean participant age of 15 years or older (~high school),^41^, ^42^, ^43^, ^44^, ^45^, ^46^ and 1/25 including roughly equal numbers of elementary and middle school students.^47^ The majority of studies included both males and females (21/25). Four studies either only included one gender or conducted separate, tailored interventions for male and female participants.^22^, ^23^, ^24^, ^40^, ^41^ Most studies were conducted among underserved populations. Specifically, 20/25 studies had a majority of participants who identified as Black, Indigenous, or person of colour and of the 11 studies that reported income, 10 included a majority of participants with low income. Eleven out of 25 studies had a weight‐based inclusion criteria to participate in the study,^24^, ^28^, ^30^, ^39^, ^40^, ^41^, ^42^, ^43^, ^44^, ^45^, ^46^ 5/25 studies did not have a weight‐based inclusion criteria to participate in the study, but analysis was limited to participants with overweight or obesity,^32^, ^33^, ^34^, ^35^ and 10/25 studies reported a subgroup analysis of participants with overweight or obesity.^22^, ^23^, ^25^, ^26^, ^27^, ^29^, ^31^, ^36^, ^37^, ^38^, ^47^

Table 2 provides a description of each study and the main weight‐based outcomes reported in each article. Overall, 6/25 studies were described as pilot or feasibility trials,^24^, ^28^, ^41^, ^42^, ^43^, ^44^ and 4/26 articles only reported follow‐up or secondary endpoint results (i.e. results at the primary endpoint were reported in a paper published prior to 2010).^23^, ^32^, ^33^, ^37^ Most studies (15/25) were randomized at the school level. Almost all studies (21/25) were two arm trials, but three studies had three arms,^29^, ^46^, ^47^ and one had four arms.^34^ For most studies the control/comparison condition was either a true control/waitlist control (5/25)^25^, ^29^, ^31^, ^34^, ^47^ or treatment as usual condition (7/25).^22^, ^23^, ^28^, ^38^, ^40^, ^42^, ^43^, ^46^ Other studies had an education only comparison (7/25)^27^, ^30^, ^32^, ^33^, ^41^, ^44^, ^45^ or a control group that provided unrelated resources to participants (1/25).^26^ Three of the 25 studies included an active comparison group^24^, ^35^, ^39^ and two studies did not provide details regarding the comparison group.^36^, ^37^

The majority (13/25) of studies did not report the theoretical underpinning of the intervention. Of the 12 studies that did report theory, Social Cognitive Theory was reported in seven studies.^22^, ^23^, ^24^, ^34^, ^38^, ^44^, ^45^, ^46^ Other reported theories included Transtheoretical Model,^42^ Self‐determination Theory,^22^, ^23^ Information Motivation Behavioural Skills Theory,^41^ and Social Ecological Models.^30^, ^31^ Most studies directly intervened with both nutrition and physical activity behaviours (12/25). Two studies only addressed nutrition or eating behaviours^31^, ^41^ and five primarily focused on physical activity.^22^, ^23^, ^24^, ^38^, ^40^, ^46^ Four studies included individual school‐based health clinic visits focused on goal setting, motivational interviewing, and other behaviour change techniques.^42^, ^43^, ^44^, ^45^ One of these school‐based health clinic studies also incorporated group physical activity sessions.^45^ Two studies did not provide direct intervention with youth: One study utilized BMI screening procedures,^47^ and the other solely provided materials and technical assistance to school staff.^27^ Intervention duration ranged from 5 weeks^37^ to 3 years,^36^ with the majority (11/25) of studies lasting 6 months^32^, ^33^, ^34^, ^35^, ^38^ to an academic year (~8–10 months).^25^, ^30^, ^31^, ^42^, ^43^, ^45^

Improved weight‐based outcomes among the intervention group compared to the control group were reported in 16/26 articles. Table 3 provides information regarding the inclusion of intervention response as an evaluation metric. Response was reported in 19% (5/26) of articles.^32^, ^33^, ^34^, ^35^, ^43^ Response was defined in three ways: maintenance or decrease in zBMI at 6 months and 1 and 2 years,^32^, ^33^, ^35^ decrease in zBMI of ≥0.10 at ~10 months,^43^ and a decrease in zBMI of ≥0.20 at 1 year.^34^ A few articles included the change in weight classification as an evaluation metric.^22^, ^23^, ^31^, ^36^, ^39^ Notably 4/5 of the articles including this metric did not report a beneficial mean intervention effect among participants with overweight or obesity. Of the five studies that reported response, three statistically compared the proportion of participants who met the threshold for response between intervention conditions.^32^, ^33^, ^43^

## 4 DISCUSSION

The results of this scoping review identified a paucity of school‐based obesity interventions that included intervention response rates. The 19% (5/26) of articles that reported intervention response rates found in this review may inflate the relative prevalence of response reporting because four^32^, ^33^, ^34^, ^35^ of the five studies^32^, ^33^, ^34^, ^35^, ^43^ that reported response were from the same research group. Consistent with secondary analyses of clinically‐based intervention,^10^, ^11^, ^12^, ^13^, ^14^, ^15^, ^16^, ^17^, ^18^ multiple definitions for response were used among school‐based interventions. Notably, only one study provided a rationale for the used response definition,^34^ which is consistent with analyses among clinic‐based interventions which also often do not provide a rational for response definition. The lack of reporting response coupled with the lack of rationale for response definitions used highlights an important gap in current evaluation of paediatric obesity treatment.

The infrequent reporting of response among school‐based obesity interventions may be due to a lack of consensus in a definition for response to paediatric obesity intervention. Few of the studies in this review defined a weight goal for the intervention,^27^, ^35^, ^45^ and only one study explained this goal prior to reporting results.^45^ This observation indicates that the a priori weight goals for school‐based obesity interventions is often to improve weight‐related outcomes more than doing nothing (control group). While this is a first step, the dissemination of programs that are “better than nothing” (i.e., have statistically significant improvements compared to control) but do not achieve meaningful reductions in weight outcomes among a substantial proportion of participants is unlikely to impact the prevalence of obesity. In the context of community‐based and public health settings, a priori intervention goals are important to determine which interventions merit dissemination, use of resources, and to evaluate intervention impact when a control group is not available. Establishing specific goals for an intervention and tracking the proportion of participants who meet the goal would provide a meaningful mechanism to continue the evaluation of intervention programs in the absence of a control group to ensure they are still beneficial.

Defining response to paediatric obesity intervention is an important and complex area in need of further research. The adult definition for response is a 5%–10% weight loss because this threshold is associated with cardiometabolic improvements. The threshold of weight‐related improvements associated with cardiometabolic improvements among youth is more complicated to determine because they are still growing and developing and because they may not have yet lived long enough for the adverse cardiometabolic consequences of obesity to have developed. Although the prevalence of elevated cardiometabolic health markers among youth has risen alongside the prevalence of obesity, many children with obesity do not yet have elevated cardiometabolic health markers.^48^, ^49^, ^50^, ^51^ Hormonal changes during puberty, a time when changes in weight and body composition naturally occur for youth, also make it difficult to define metabolic syndrome in youth.^52^, ^53^, ^54^ Furthermore, the effects of having excess adiposity during puberty differ between individuals with and without other cardiometabolic risk factors.^55^ Finally, the magnitude of weight changes needed to improve cardiometabolic risk varies depending on the particular marker of cardiometabolic health examined.^56^, ^57^, ^58^, ^59^, ^60^, ^61^ Due to these complicated factors, the United States Preventive Task Force was unable to identify a specific threshold of weight improvement associated with a cardiometabolic improvements, but generally agreed with European researchers that a 0.20–0.25 zBMI reduction is likely to have meaningful health benefits.^8^, ^62^ The Endocrine Society identified a 1.5 kg/m^2^ decrease in BMI as of important benefit to youth with overweight and obesity and a 7% decrease in weight as a realistic goal for youth with severe obesity.^63^

Notably, the duration of an intervention and length of follow‐up are important factors in defining realistic a priori weight goals. Definitions for response varied by study duration and time of follow‐up. For example, 0.1 decrease in zBMI was used at 10 months (at the end of the intervention), whereas a decrease of 0.2 was used at a year (which was at 6‐month follow‐up). If response is defined as a set amount of change, the amount of weight loss should be realistic for the time frame evaluated. Conversely, the response definition of a decrease or maintenance in zBMI was used at a variety of timepoints including 6 months (at the end of the intervention), 1 year (at 6‐month follow‐up), and 2 years (1.5 year follow‐up). While the rationale for the reasoning behind this definition was not discussed, this type of response definition appears to emphasize the importance of preventing further weight gain, rather than being concerned with a set amount of improvement. Given the challenges of weight maintenance following intervention, a priori goals for follow‐up assessment may also need to differ from the primary endpoint of the intervention. Research regarding how to meaningfully define response to paediatric obesity intervention is clearly needed. Given the complexity of understanding the relationship between weight and cardiometabolic outcomes among youth, it is possible that alternative metrics need to be considered to define meaningful response besides the weight loss needed to see cardiometabolic improvement.

The primary reason for article exclusion was because weight‐related outcomes were not reported separately for participants classified with overweight or obesity from those with a healthy weight classification (57% of excluded articles were excluded because analyses were among a population of youth with a mix weight statuses). A unique challenge of school‐based obesity intervention is that interventions often include children of all weight classifications. The inclusion of students of all weight statuses prevents potential stigmatization and is feasible because behaviours promoted are similar for both primary and secondary prevention of obesity. However, because the youth of varying weight classifications inherently have different weight‐related goals, the definition of response to intervention needs to vary by weight classification. Analysing the weight related outcomes of all participants together regardless of weight status is difficult to interpret and prevents understanding for how school‐based interventions may contribute to the secondary prevention of obesity.

This study is the first to examine response definitions and reporting among school‐based obesity interventions. This study has been reported according to the PRISMA‐ScR, which lends strength to the methodology. Additionally, the inclusion of five databases covering biomedicine, psychology, and education helped to ensure a comprehensive search was conducted for this interdisciplinary topic. Limitations of this review include that the protocol was not registered prior to the study being carried out and the authors were not contacted for more details. Additionally, research records written in languages other than English were excluded due to a lack of resources for translation. Lastly, although helpful for comparison across relatively similar schooling systems, the exclusion of non‐westernized nations limits the generalizability of findings. Generalizability of findings may also be limited by including only randomized control trials published after 2010 as it is possible these inclusion criteria may have resulted in missed articles. For example, practicalities of doing research in the school setting can preclude researchers from using a randomized control trial design.

## 5 CONCLUSIONS

Behavioural lifestyle intervention is the cornerstone of obesity prevention and treatment. Interventions in community settings like schools can reach diverse populations and help facilitate healthy behaviour change. School‐based interventions also face a number of practicalities that can limit the scope of interventions provided (e.g., level of teacher training, academic priorities, limited resources, overburdened staff). It is important that the evaluation of school‐based obesity interventions be conducted in a manner that clearly informs decisions regarding the next steps and dissemination. Results of the present scoping review indicate that response is rarely included as an evaluation metric among school‐based interventions. Including response is an important step to better understand which interventions are most beneficial for whom, to inform decisions regarding intervention dissemination, and to continue rigorously evaluation of interventions once implemented into practice. The articles included in this review illustrate the great heterogeneity in intervention types that occur in school settings as well as the diversity of populations served in schools. Having a set definition for meaningful response is particularly critical to help compare effectiveness across the wide range of populations and intervention types aimed at addressing obesity, especially when interventions are evaluated with various study designs. As the proportion of participants responsive to an intervention can vary greatly depending on the criteria for response, researchers are encouraged to select a response definition or intervention goal a priori.

## AUTHOR CONTRIBUTIONS

KA conceived the premise of the paper and wrote the initial draft. RH developed and conducted the searches. AC, CJ, KA, and TL screened and extracted data from articles. LG, AC, CJ, and TL provided critical scientific review of the manuscript. All authors had final approval of the submitted and published versions.

## CONFLICT OF INTEREST

None of the authors have any conflicts of interest to report.

## Supporting information


**Appendix S1** Supplementary Information.
