# Supplementary material for: Response to school‐based interventions for overweight and obesity: A systematic scoping review
Source: Clin Obes. 2022 Sep 21;12(6):e12557. doi: 10.1111/cob.12557 (PMC9669238; doi:10.1111/cob.12557)
Supplement: Supplementary file 1 — Appendix S1 Supplementary Information. [file COB-12-e12557-s001.pdf]

APPENDIX A. Search Strategy

PubMed:
("school health services"[mesh] OR “school health”[tiab] OR “school based”[tiab] OR “after school”[tiab] OR “school program”[tiab] OR "physical education and training"[mesh] OR “physical education”[tiab] OR “school nursing”[mesh] OR school nurs*[tiab] OR school setting*[tiab]) AND ("body weight"[mesh:noexp] OR “body weight”[tiab] OR "body weight changes"[mesh:noexp] OR "body composition"[mesh:noexp] OR overweight[mesh] OR overweight[tiab] OR obesity[mesh] OR obesity[tiab] OR “pediatric obesity”[mesh] OR thinness[mesh] OR thinness[tiab] OR “ideal body weight”[mesh] OR "ideal body weight"[tiab] OR "weight loss"[mesh:noexp] OR “weight loss”[tiab] OR weightloss[tiab] OR "body weight maintenance"[mesh] OR “body weight maintenance”[tiab] OR “body mass index”[mesh] OR "body mass index"[tiab] OR “body mass indices”[tiab] OR BMI[tiab] OR zBMI[tiab] OR “z BMI”[tiab] OR BMIz[tiab] OR “BMI z”[tiab]) AND (adolescent[mesh] OR adolescen*[tiab] OR teenage*[tiab] OR teen[tiab] OR teens[tiab] OR child[mesh] OR child*[tiab] OR “elementary aged”[tiab] OR “elementary school”[tiab] OR “elementary schools”[tiab] OR “primary school”[tiab] OR “primary schools”[tiab] OR “middle school”[tiab] OR “middle schools”[tiab] OR “junior high”[tiab] OR “junior highs”[tiab] OR “junior high school”[tiab] OR “junior high schools”[tiab] OR “high school”[tiab] OR “high schools”[tiab] OR “secondary school”[tiab] OR “secondary schools”[tiab] OR “secondary education”[tiab]) AND (“randomized controlled trial”[pt] OR “controlled clinical trial”[pt] OR randomized[tiab] OR placebo[tiab] OR “clinical trials as topic”[mesh:noexp] OR randomly[tiab] OR trial[ti] NOT (animals[mesh] NOT humans[mesh])) NOT (africa[mesh] OR "antarctic regions"[mesh] OR "arctic regions"[mesh] OR asia[mesh])

Filters: 2010 - present; English

Scopus:
TITLE-ABS-KEY("school health services" OR “school health” OR “school based” OR “after school” OR “school program” OR "physical education and training" OR “physical education” OR “school nursing” OR school nurs* OR school setting*) AND TITLE-ABS-KEY("body weight" OR "body weight changes" OR "body composition" OR overweight OR obesity OR “pediatric obesity” OR thinness OR “ideal body weight” OR "weight loss" OR weightloss OR "body weight maintenance" OR “body mass index” OR “body mass indices” OR BMI OR zBMI OR “z BMI” OR BMIz OR “BMI z”) AND TITLE-ABS-KEY(adolescent OR adolescen* OR teenage* OR teen OR teens OR child OR child* OR “elementary aged” OR “elementary school” OR “elementary schools” OR “primary school” OR “primary schools” OR “middle school” OR “middle schools” OR “junior high” OR “junior highs” OR “junior high school” OR “junior high schools” OR “high school” OR “high schools” OR “secondary school” OR “secondary schools” OR “secondary education”) AND TITLE-ABS-KEY(“randomized controlled trial” OR “controlled clinical trial” OR randomized OR placebo OR “clinical trials as topic” OR randomly OR trial) AND NOT TITLE-ABS-KEY(animals AND NOT humans) AND NOT TITLE-ABS-KEY(africa OR "antarctic regions" OR "arctic regions” OR asia)

Filters: 2010 - present; English; Article or Review

PsycInfo (EBSCO):
("school health services" OR “school health” OR “school based” OR “after school” OR “school program” OR "physical education and training" OR “physical education” OR “school nursing” OR school nurs* OR school setting*) AND (“body weight” OR "body weight changes" OR "body composition" OR overweight OR obesity OR “pediatric obesity” OR thinness OR "ideal body weight" OR “weight loss” OR weightloss OR “body weight maintenance” OR "body mass index" OR “body mass indices” OR BMI OR zBMI OR “z BMI” OR BMIz OR “BMI z”) AND (adolescent OR adolescen* OR teenage* OR teen OR teens OR child OR child* OR “elementary aged” OR “elementary school” OR “elementary schools” OR “primary school” OR “primary schools” OR “middle school” OR “middle schools” OR “junior high” OR “junior highs” OR “junior high school” OR “junior high schools” OR “high school” OR “high schools” OR “secondary school” OR “secondary schools” OR “secondary education”) AND (“randomized controlled trial” OR “controlled clinical trial” OR randomized OR placebo OR “clinical trials as topic” OR randomly OR trial NOT (animals NOT humans)) NOT (africa OR “antarctic regions” OR “arctic regions” OR asia)

Filters: 2010 - present; English; Academic Journals

Education Source (EBSCO):
("school health services" OR “school health” OR “school based” OR “after school” OR “school program” OR "physical education and training" OR “physical education” OR “school nursing” OR school nurs* OR school setting*) AND (“body weight” OR "body weight changes" OR "body composition" OR overweight OR obesity OR “pediatric obesity” OR thinness OR "ideal body weight" OR “weight loss” OR weightloss OR “body weight maintenance” OR "body mass index" OR “body mass indices” OR BMI OR zBMI OR “z BMI” OR BMIz OR “BMI z”) AND (adolescent OR adolescen* OR teenage* OR teen OR teens OR child OR child* OR “elementary aged” OR “elementary school” OR “elementary schools” OR “primary school” OR “primary schools” OR “middle school” OR “middle schools” OR “junior high” OR “junior highs” OR “junior high school” OR “junior high schools” OR “high school” OR “high schools” OR “secondary school” OR “secondary schools” OR “secondary education”) AND (“randomized controlled trial” OR “controlled clinical trial” OR randomized OR placebo OR “clinical trials as topic” OR randomly OR trial NOT (animals NOT humans)) NOT (africa OR “antarctic regions” OR “arctic regions” OR asia)

Filters: 2010 - present; English; Academic Journals

ERIC (EBSCO):
("school health services" OR “school health” OR “school based” OR “after school” OR “school program” OR "physical education and training" OR “physical education” OR “school nursing” OR school nurs* OR school setting*) AND (“body weight” OR "body weight changes" OR "body composition" OR overweight OR obesity OR “pediatric obesity” OR thinness OR "ideal body weight" OR “weight loss” OR weightloss OR “body weight maintenance” OR "body mass index"

OR “body mass indices” OR BMI OR zBMI OR “z BMI” OR BMIz OR “BMI z”) AND (adolescent OR adolescen* OR teenage* OR teen OR teens OR child OR child* OR “elementary aged” OR “elementary school” OR “elementary schools” OR “primary school” OR “primary schools” OR “middle school” OR “middle schools” OR “junior high” OR “junior highs” OR “junior high school” OR “junior high schools” OR “high school” OR “high schools” OR “secondary school” OR “secondary schools” OR “secondary education”) AND (“randomized controlled trial” OR “controlled clinical trial” OR randomized OR placebo OR “clinical trials as topic” OR randomly OR trial NOT (animals NOT humans)) NOT (africa OR “antarctic regions” OR “arctic regions” OR asia)

Filters: 2010 - present; English; Academic Journals
